# Analysis of data for comorbidity and survival in out-of-hospital cardiac arrest

**DOI:** 10.1016/j.dib.2018.11.010

**Published:** 2018-11-06

**Authors:** Geir Hirlekar, Martin Jonsson, Thomas Karlsson, Jacob Hollenberg, Per Albertsson, Johan Herlitz

**Affiliations:** a Department of Cardiology, Sahlgrenska University Hospital, Gothenburg, Sweden; b Karolinska Institutet, Department of Medicine, Centre for Resuscitation Science, Stockholm, Sweden; c Health Metrics Unit, Institute of Medicine, Sahlgrenska Academy at University of Gothenburg, Gothenburg, Sweden; d Faculty of Caring Science,Work Life and Social Welfare, University of Borås, Sweden; e Prehospital-Centre for Prehospital Research,Work Life and Social Welfare, University of Borås, Sweden

## Abstract

The data presented in this article is supplementary to the research article titled ”Comorbidity and survival in out-of-hospital cardiac arrest” (Hirlekar et al., 2018).

The data contains information of how Charlson Comorbidity Index (CCI) is calculated and coded from ICD-10 codes. Multivariable logistic regression was used in the analysis of association between comorbidity and return of spontaneous circulation. We present baseline characteristics of patients found in VF/VT. All patients with non-missing data on all baseline variables are analyzed separately. We compare the baseline characteristics of patients with and without complete data set. Analysis of when comorbidity was identified in relation to outcome is also shown.

**Specifications table**

**Table:** 

Subject area	*Cardiac arrest.*
More specific subject area	*Epidemiology of cardiac arrest.*
Type of data	*Tables and figures.*
How data was acquired	*Data analysis from the National Patient Registry (NPR) and the Swedish Registry of Cardiopulmonary Resuscitation (SRCR).*
Data format	*Analyzed.*
Experimental factors	*Data was analyzed to investigate whether comorbidity is associated with outcome in out-of-hospital cardiac arrest (OHCA).*
Experimental features	*Nationwide retrospective and population-based cohort study of patients with bystander witnessed OHCA.*
Data source location	*A nationwide cohort study in Sweden.*
Data accessibility	*The analyzed data are presented in this article.*
Related research article	*G. Hirlekar, M. Jonsson, T. Karlsson et al. Comorbidity and survival in out-of-hospital cardiac arrest. In press.*

**Value of the data**

• The data provides information about how ICD-10 codes were used to create the categories in Charlson Comorbidity Index (CCI).
• The data provides information of association between comorbidity and return of spontaneous circulation (ROSC).
• The data provides information of the baseline characteristics of patients found in VF/VT.
• The data provides comparison of patients with complete data and patients with missing data.
• The data shows association between comorbidity and survival depending on when the comorbidity condition was identified.

## 1 Data

**Fig. 1:**
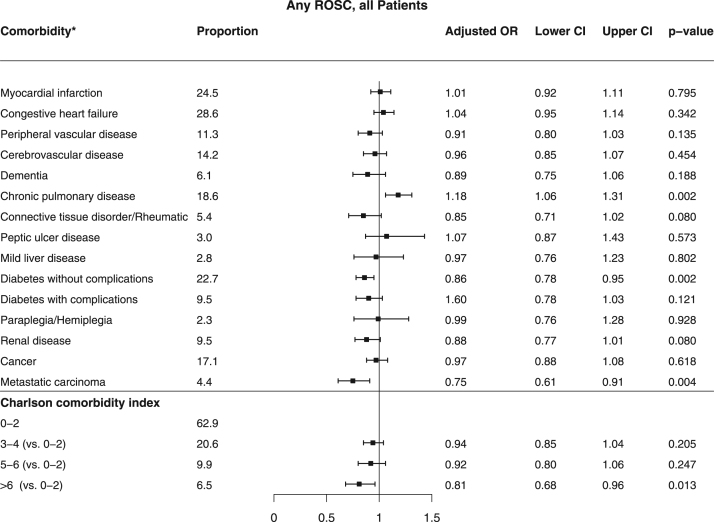
Patients with any ROSC and relation to comorbidity (*n*=4,612). * The comorbidities of moderate or severe liver disease and AIDS/HIV were not analyzed in the specific comorbidity conditions above, due to low prevalence (0.6% and 0.1%, respectively).

**Fig. 2:**
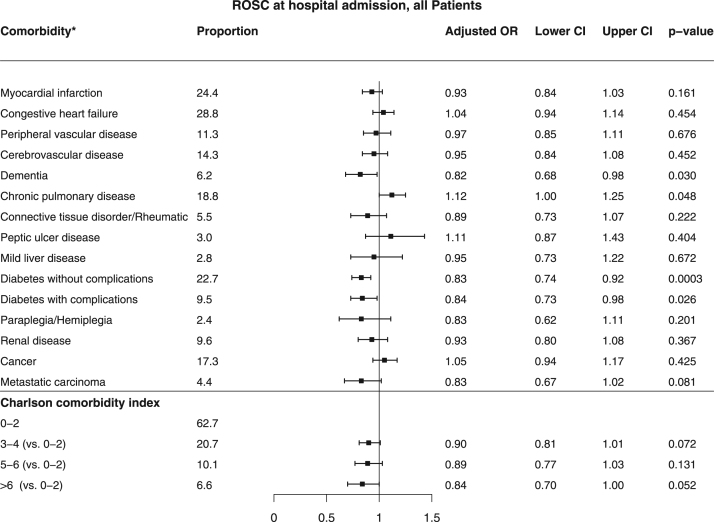
Patients with ROSC at hospital admission and relation to comorbidity (*n*=3,690). * The comorbidities of moderate or severe liver disease and AIDS/HIV were not analyzed in the specific comorbidity conditions above, due to low prevalence (0.6% and 0.1%, respectively).

**Fig. 3:**
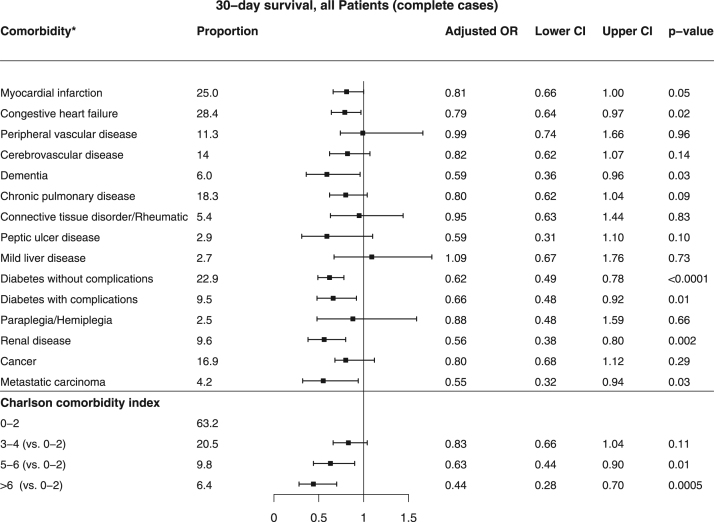
Patients with non-missing data on all baseline characteristics (*n*=8,193). (1014 (12.4%) patients alive at 30 days). * The comorbidities of moderate to severe liver disease and AIDS/HIV were not analyzed in the specific comorbidity conditions above, due to low prevalence (0.6% and 0.0%, respectively).

**Fig. 4:**
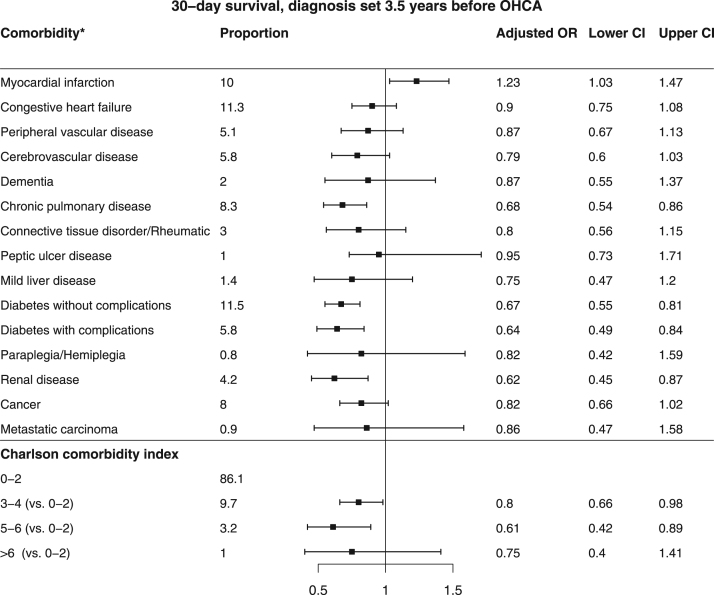
Patients for whom comorbidity conditions were identified 3–5 years before OHCA. * The comorbidities of moderate or severe liver disease and AIDS/HIV were not analyzed in the specific comorbidity conditions above, due to low prevalence (0.2% and 0.1%, respectively).

**Fig. 5:**
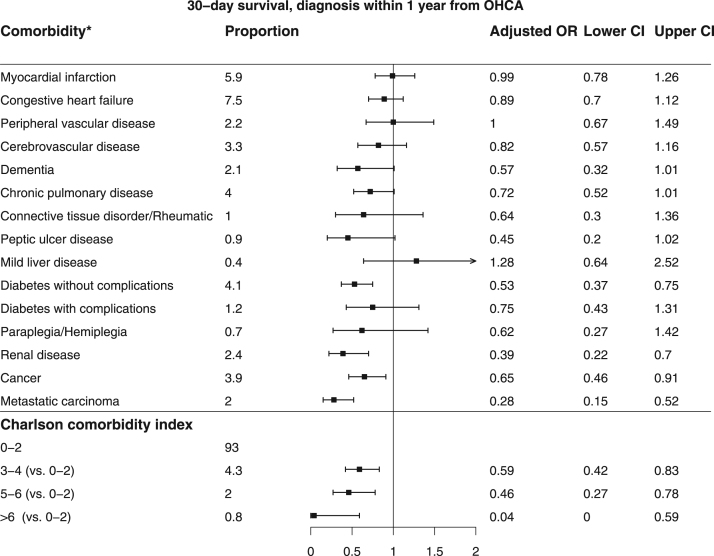
Patients for whom comorbidity was first identified within 1 year before OHCA. * The comorbidities of moderate or severe liver disease and AIDS/HIV were not analyzed in the specific comorbidity conditions above, due to low prevalence (0.2% and 0.0%, respectively).

**Fig. 6:**
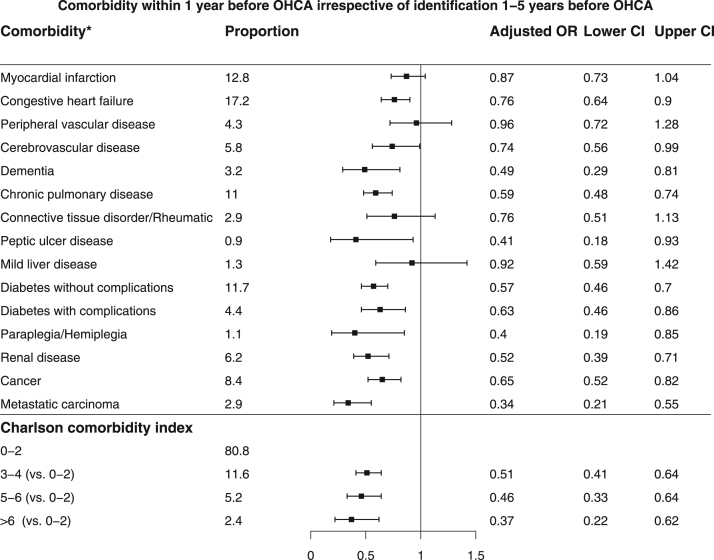
Comorbidity conditions within 1 year before OHCA irrespective of identification 1–5 years before OHCA. * The comorbidities of moderate or severe liver disease and AIDS/HIV were not analyzed in the specific comorbidity conditions above, due to low prevalence (0.3% and 0.0%, respectively).

The data contains information of how ICD-10 codes were used to create the categories in Charlson Comorbidity Index (CCI) as shown in Table 1 [2]. Baseline characteristics of patients found in VF/VT are shown in Table 2. Comparison of baseline characteristics of cases with and without complete data are shown in Table 3. The association between missingness and other baseline characteristics, CCI and survival for all patients are shown in Table 4. The relation between comorbidity and the chance of any return of spontaneous circulation (ROSC) is shown in Fig. 1 and the corresponding figure for ROSC at hospital admission is shown in Fig. 2. Association between various aspects of comorbidity and 30-day survival among all patients with complete cases on all baseline characteristics (no missing) is shown in Fig. 3. In Fig. 4, Fig. 5, Fig. 6, we present the association between comorbidity and 30-day survival in relation to the time of identification of the comorbidity condition, as follows: Patients for whom comorbidity condition were identified 3–5 years before OHCA (Fig. 4); first identified within 1 year before OHCA (Fig. 5); and comorbidity condition within 1 year before OHCA irrespective of identification 1–5 years before OHCA (Fig. 6).

**Table 1:** Charlson comorbidity index according to ICD-10 codes.

**Disease**	**ICD 10**	**Weight point**
*Myocardial infarction*	(I21.x, I22.x, I25.2),	1
*Congestive heart failure*	(I09.9, I11.0, I13.0, I13.2, I25.5, I42.0, I42.5–I42.9, I43.x, I50.x, P29.0)	1
*Peripheral vascular disease*	(I70.x, I71.x, I73.1, I73.8, I73.9, I77.1, I79.0, I79.2, K55.1, K55.8, K55.9, Z95.8, Z95.9)	1
*Cerebrovascular disease*	(G45.x, G46.x, H34.0, I60.x–I69.x)	1
*Dementia*	(F00.x–F03.x, F05.1, G30.x, G31.1)	1
*Chronic pulmonary disease*	(I27.8, I27.9, J40.x–J47.x, J60.x–J67.x, J68.4, J70.1, J70.3)	1
*Rheumatic disease*	(M05.x, M06.x, M31.5, M32.x–M34.x, M35.1, M35.3, M36.0)	1
*Peptic ulcer disease*	(K25.x–K28.x)	1
*Mild liver disease*	(B18.x, K70.0–K70.3, K70.9, K71.3–K71.5, K71.7, K73.x, K74.x, K76.0, K76.2–K76.4, K76.8, K76.9, Z94.4)	1 (0 if also moderate or severe liver disease)
*Diabetes without chronic complications*	(E10.0, E10.1, E10.6, E10.8, E10.9, E11.0, E11.1, E11.6, E11.8, E11.9, E12.0, E12.1, E12.6, E12.8, E12.9, E13.0, E13.1, E13.6, E13.8, E13.9, E14.0, E14.1, E14.6, E14.8, E14.9)	1 (0 if also diabetes with chronic complications)
*Diabetes with chronic complications*	(E10.2–E10.5, E10.7, E11.2–E11.5, E11.7, E12.2–E12.5, E12.7, E13.2– E13.5, E13.7, E14.2–E14.5, E14.7)	2
*Hemiplegia/paraplegia*	(G04.1, G11.4, G80.1, G80.2, G81.x, G82.x, G83.0–G83.4, G83.9)	2
*Renal disease*	(I12.0, I13.1, N03.2–N03.7, N05.2– N05.7, N18.x, N19.x, N25.0, Z49.0– Z49.2, Z94.0, Z99.2)	2
*Cancer*	(C00.x–C26.x, C30.x–C34.x, C37.x– C41.x, C43.x, C45.x–C58.x, C60.x– C76.x, C81.x–C85.x, C88.x, C90.x–C97.x)	2 (0 if also metastatic carcinoma)
Moderate or severe liver disease	(I85.0, I85.9, I86.4, I98.2, K70.4, K71.1, K72.1, K72.9, K76.5, K76.6, K76.7)	3
*Metastatic carcinoma*	(C77.x–C80.x)	6
*AIDS/HIV*	(B20.x–B22.x, B24.x)	6
**Maximum possible score**		**29**

**Table 2:** Baseline characteristics of VF/VT patients.

	**All patients(*n*=3,468)**	**Alive at 30 days**		***p*-value**
		**Yes**	**No**	
		**(*n*=1,098)**	**(*n*=2,370)**	
**Year of OHCA:**				0.03*
2011	693 - 20.0	210 - 19.1	483 - 20.4	
2012	696 - 20.1	202 - 18.4	494 - 20.8	
2013	676 - 19.5	216 - 19.7	460 - 19.4	
2014	671 - 19.3	214 - 19.5	457 - 19.3	
2015	732 - 21.1	256 - 23.3	476 - 20.1	
**OHCA during daytime 8 a.m. to 8 a.m.** (87/181)**	2,281 - 71.3	773 - 76.5	1,508 - 68.9	<0.0001
**Age, years**	69 (50,84)	64 (45,79)	71 (54,86)	<0.0001
**Female sex**	656 - 18.9	200 - 18.2	456 - 19.2	0.48
**OHCA at home** (2/0)	2,020 - 58.3	464 - 42.3	1,556 - 65.7	<0.0001
**CPR before arrival of EMS** (5/7)	2,784 - 80.6	953 - 87.2	1,831 - 77.5	<0.0001
**Mechanical chest compression** (83/144)	1,313 - 40.5	290 - 28.6	1,023 - 46.0	<0.0001
**Cardiac aetiology** (50/115)	2,830 - 85.7	898 - 85.7	1,932 - 85.7	1.00
**Treatment:**				
Adrenalin (33/15)	2,734 - 79.9	532 - 50.0	2,202 - 93.5	<0.0001
Intubation (18/31)	1,271 - 37.2	274 - 25.4	997 - 42.6	<0.0001
Anti-arrhythmics (45/60)	1,394 - 41.5	264 - 25.1	1,130 - 48.9	0.01
Defibrillation (84/63)	3,317 - 99.9	1,013 - 99.9	2,304 - 99.9	1.00
No. of defibrillations^c^	3 (1,9)	2 (1,8)	4 (1,10)	<0.0001
**Delay, minutes:**				
Collapse to start of CPR (131/265)	2 (0,12)	1 (0,8)	3 (0,15)	<0.0001
Collapse to first defibrillation^c^ (115/248)	13 (6,24)	11 (5,19)	14 (7,26)	<0.0001
Call for EMS to EMS arrival (199/346)	8 (4,19)	7 (3,15)	9 (4,20)	<0.0001
**Survival at 30 days**	1,098–31.7	1,098 - 100	0 - 0	

Data are presented as number - percentage (%) or median (10th, 90th percentile).

* Year of OHCA as an ordered variable.

** Number of patients with missing information (of those alive/not alive at 30 days).

c Of those defibrillated (*n*=1,013/2,304).

**Table 3:** Comparison of patients with and without complete data.

	**All patients (*n*=12012)**	**Complete data**		***p* value**
		**Yes (*n*=8193)**	**No (*n*=3819)**	
**Year of OHCA**				<0.0001#
2011	19.1	16.8	23.8	
**2012**	**19.0**	**18.6**	**19.8**	
**2013**	**20.3**	**20.6**	**19.7**	
**2014**	**20.0**	**19.8**	**20.4**	
**2015**	**21.6**	**24.1**	**16.3**	
**OHCA during daytime 08–20** (941)*	66.0	65.0	68.8	0.0002
**Age** (years)	72 (52,88)	72 (52,87)	72 (51,88)	0.92
**Female sex**	31.7	31.6	31.8	0.90
**VF/VT as initial arrhythmia** (433)	30.0	30.1	29.5	0.50
**OHCA at home** (7)	70.6	71.7	68.0	<0.0001
**CPR before arrival of EMS** (57)	71.2	72.2	69.1	0.0006
**Mechanical chest compression** (881)	37.7	41.3	27.8	<0.0001
**Cardiac etiology** (561)	70.7	70.2	72.1	0.04
**Treatment**				
Adrenalin (142)	83.0	84.7	79.1	<0.0001
Intubation (104)	36.6	33.4	43.7	<0.0001
Anti-arrhythmics (283)	16.2	17.4	13.5	<0.0001
**Delay** (minutes)				
Collapse to start of CPR (1453)	4 (0,16)	3 (0,15)	5 (0,19)	<0.0001
Call for EMS to EMS arrival (2084)	10 (4,21)	10 (4,21)	10 (4,22)	0.46
**Survival at 30 days**				
All patients	13.3	12.4	15.3	<0.0001
Patients found in ventricular fibrillation	31.7	31.2	32.8	0.35
Patients with other initial arrhythmia	4.2	4.3	3.9	0.54

Results presented as percentage (%) or median (10th, 90th percentile).

#Year of OHCA as an ordered variable.

*Number of patients with missing information.

**Table 4:** Associations between missingness and other baseline characteristics, CCI and survival in all patients.

Missingness of	Baseline characteristics															
	Year of OHCA	OHCA during day-time	Age	Sex	Initial rhythm	OHCA at home	CPR before arrival of EMS	Mechanical CC	Cardiac aeti-logy	Adrenalin	Intubation	Antiarrhythmics	OHCA to start of CPR	Call for EMS to arrival	CC index	Alive at 30 days
OHCA during daytime	X	NA	–	–	–	X	–	X	X	X	X	–	–	X	–	–
Initial rhythm	X	X	–	–	NA	X	X	X	X	X	X	X	X	–	–	X
OHCA at home	X	–	–	–	–	NA	–	–	–	–	–	–	–	–	–	–
CPR before arrival of EMS	–	–	–	X	–	–	NA	–	–	X	–	–	–	–	–	–
Mechanical CC	X	–	–	–	–	–	X	NA	X	–	X	X	X	–	X	–
Cardiac aetiology	X	X	–	–	X	X	–	X	NA	X	–	–	–	–	X	–
Adrenalin	–	X	–	–	–	X	–	X	–	NA	X	X	X	–	–	X
Intubation	–	–	X	–	X	X	–	X	–	X	NA	X	–	–	–	X
Anti-arrhythmics	X	X	–	–	X	X	–	–	X	X	X	NA	X	–	–	X
CA to start of CPR	X	–	–	–	–	–	X	X	X	X	–	–	NA	X	–	–
Call for EMS to EMS arrival	X	X	–	–	X	X	–	X	X	–	X	X	X	NA	X	–

X=an association found using Fisher׳s exact test or Mann-Whitney U test (*p*<0.05).

----=no association (*p*>0.05).

NA=not applicable.

## 2 Experimental design, materials, and methods

We conducted an analysis of data from the Swedish Registry for Cardiopulmonary Resuscitation (SRCR) which was collected between 2011 and 2015. We linked the data from SRCR with data from the National Patient Registry (NPR). The NPR includes data on diagnoses and surgical procedure codes from hospitals and specialist clinics [3]. We had data on health disorders during the five years preceding the OHCA. We measured comorbidity with CCI as shown in Table 1. If the patient had any mention of an ICD-10 code listed in the NPR which was included in the category definition of CCI, the patient would get a weight point with the maximum possible score of 29.

## 3 Study design

We performed a nationwide population-based cohort study of patients with bystander witnessed OHCA which was designed to evaluate if there were any association between comorbidity and outcome. We included all cases with bystander-witnessed OHCA who were ≥18 years of age. Unwitnessed and cases only witnessed by Emergency Medical Service (EMS) were excluded. For details, see Ref. [1].

## 4 Statistical analysis

We used logistic regression and made adjustments for year of OHCA, age, sex, initial rhythm, location, bystander cardiopulmonary resuscitation (CPR), mechanical chest compression, aetiology, adrenalin treatment, intubation, anti-arrhytmics, time to CPR and EMS response time. Fisher׳s exact test was used to test for difference between groups regarding dichotomous variables and Mann-Whitney U test for ordered/continuous variables in the baseline characteristics. We used multiple imputation for the multivariable analysis and the missing data were assumed to be missing at random (MAR). To exclude that the missing data pattern were missing completely at random (MCAR) we compared cases with no missing data with incomplete cases (Table 3) and found several major differences. The assumption of a MAR pattern was indicated to be valid by examination of the associations between missingness of each variable with other variables (Table 4). We analysed also complete cases without multiple imputation (Fig. 3). Outcome endpoint was not imputed and thus only patients with any ROSC or ROSC at hospital admission were included in the analysis in Fig. 1, Fig. 2.
