# Supplementary material for: Analysis of data for comorbidity and survival in out-of-hospital cardiac arrest
Source: Data Brief. 2018 Nov 6;21:1541–51. doi: 10.1016/j.dib.2018.11.010 (PMC6240633; doi:10.1016/j.dib.2018.11.010)
Supplement: Supplementary file 1 — Supplementary material. [file mmc1.docx]

Dear Editor and Co-Editors of the journal Data in Brief,

**The authors declare that they have no conflicts of interests.**

Sincerely, on behalf of all the authors,

**Geir Hirlekar, MD**

Department of Cardiology,

Sahlgrenska University Hospital,

Blå stråket 3, vån 1

4135 Gothenburg

Sweden
